# Infectious Bronchitis Virus Infection Increases Pathogenicity of H9N2 Avian Influenza Virus by Inducing Severe Inflammatory Response

**DOI:** 10.3389/fvets.2021.824179

**Published:** 2022-02-08

**Authors:** Lingchen Kong, Renrong You, Dianchen Zhang, Qingli Yuan, Bin Xiang, Jianpeng Liang, Qiuyan Lin, Chan Ding, Ming Liao, Libin Chen, Tao Ren

**Affiliations:** ^1^College of Veterinary Medicine, South China Agricultural University, Guangzhou, China; ^2^Key Laboratory of Animal Vaccine Development, Ministry of Agriculture, Guangzhou, China; ^3^National and Regional Joint Engineering Laboratory for Medicament of Zoonosis Prevention and Control, Guangzhou, China; ^4^Key Laboratory of Zoonosis Prevention and Control of Guangdong Province, Guangzhou, China; ^5^Shanghai Veterinary Research Institute (SHVRI), Chinese Academy of Agricultural Sciences (CAAS), Shanghai, China

**Keywords:** infectious bronchitis virus, H9N2 avian influenza virus, pathogenicity, inflammatory response, NLRP3, transcriptome analysis

## Abstract

Infectious bronchitis virus (IBV) and H9N2 avian influenza virus (AIV) are frequently identified in chickens with respiratory disease. However, the role and mechanism of IBV and H9N2 AIV co-infection remain largely unknown. Specific-pathogen-free (SPF) chickens were inoculated with IBV 2 days before H9N2 virus inoculation (IBV/H9N2); with IBV and H9N2 virus simultaneously (IBV+H9N2); with H9N2 virus 2 days before IBV inoculation (H9N2/IBV); or with either IBV or H9N2 virus alone. Severe respiratory signs, pathological damage, and higher morbidity and mortality were observed in the co-infection groups compared with the IBV and H9N2 groups. In general, a higher virus load and a more intense inflammatory response were observed in the three co-infection groups, especially in the IBV/H9N2 group. The same results were observed in the transcriptome analysis of the trachea of the SPF chickens. Therefore, IBV might play a major role in the development of respiratory disease in chickens, and secondary infection with H9N2 virus further enhances the pathogenicity by inducing a severe inflammatory response. These findings may provide a reference for the prevention and control of IBV and H9N2 AIV in the poultry industry and provide insight into the molecular mechanisms of IBV and H9N2 AIV co-infection in chickens.

## Introduction

Coronaviruses are important infectious pathogens affecting humans and animals and are classified into four genera: α, β, γ, and δ (1). In recent years, several coronaviruses, including severe acute respiratory syndrome coronavirus, Middle East respiratory coronavirus, and severe acute respiratory syndrome coronavirus 2 (SARS-CoV-2), have caused severe respiratory disease in humans (2). Of note, the SARS-CoV-2 has infected over 70 million people worldwide. Co-infection with SARS-CoV-2 and influenza A virus has also been reported in patients with respiratory disease (3–5). Infectious bronchitis virus (IBV) is a gammacoronavirus with an ~27-kb positive-sense single-stranded RNA genome that encodes four structural proteins, including the spike glycoprotein and the envelope, membrane, and nucleocapsid proteins, as well as four non-structural accessory proteins, 3a, 3b, 5a, and 5b (6). It mainly damages the upper respiratory tract, although some strains can induce pathological changes in the kidneys and reproductive tract, leading to a drop in egg production and an increased mortality rate in chickens (7–10). The RNA-dependent RNA polymerase of IBV has limited proofreading ability, resulting in a variety of serotypes (11, 12). To control IBV infection, commercial vaccines have been widely used in poultry. However, IBV outbreaks have been reported frequently because of the limited cross-protection of the vaccines (13, 14).

Respiratory disease in chickens is a common cause of high mortality in poultry, posing a great impact on the poultry industry worldwide (15–17). Previous studies have demonstrated that co-infection with multiple respiratory pathogens, including IBV, avian influenza virus (AIV) (2), Newcastle disease virus, *Mycoplasma gallisepticum*, avian metapneumovirus, and infectious laryngotracheitis virus, is common in chickens (16, 18, 19). It is reported that co-infection with multiple respiratory pathogens could increase the morbidity and mortality rates (16, 20, 21). H9N2 AIV is widespread in poultry worldwide and has become one of the dominant AIV subtypes in chicken flocks in China, resulting in great economic losses to poultry production (22, 23). Although H9N2 AIV is a low-pathogenicity virus, co-infection with other pathogens can induce severe clinical disease and a high mortality rate (24, 25). Moreover, H9N2 AIV provide partial or even whole set of internal genes to emerging human-lethal H5N1, H7N9, H10N8 and H5N6 reassortants, posing a substantial threat to public health (26–29). Two of the most common respiratory pathogens, IBV and H9N2 AIV, are often seen as mixed infections in poultry. Hassan et al. (18) screened 86 broiler flocks with respiratory diseases from January 2012 to February 2014 in four provinces in Egypt for respiratory virus pathogens and found that mixed infections accounted for 66.3% of infections in the tested chickens and that mixed infections with IBV and H9N2 AIV were the most common (41.7%). Roussan et al. (19) tested the tracheal samples of 115 commercial broilers with respiratory diseases, and the results showed that both IBV and H9N2 AIV could be detected in 15.7% of samples. Some studies have shown that co-infection of IBV with AIV could produce severe clinical outcomes, gross lesions, and high mortality rates (20, 30, 31). However, some studies have demonstrated viral interference between the H9N2 virus and IBV vaccine strains *in vitro*, in ovo, and *in vivo* (32, 33). The virulence of the IBV and H9N2 viruses and the immune response of the host may affect the clinical manifestation of co-infection. However, the immune response to and the pathogenicity of co-infection of IBV and H9N2 AIV circulating in China remain largely unknown.

In this study, in order to provide a better theoretical basis for prevention and control of co-infection with IBV and H9N2, we systemically investigated the effect of co-infection with wild-type IBV and H9N2 AIV, which are epidemic in China, on pathogenicity in a specific-pathogen-free (SPF) chicken model and found that IBV could increase the pathogenicity of H9N2 AIV. We also demonstrated that co-infection with IBV and H9N2 virus could exacerbate inflammation in the trachea, lung, and kidneys. Furthermore, the transcriptome analysis of the trachea of the SPF chickens showed that co-infection with IBV and H9N2 virus could induce a stronger inflammatory response. These findings expand our understanding of the synergistic infection of IBV and H9N2 viruses in chickens.

## Materials and Methods

### Ethics Statement

All animal experiments involved in this study were approved by the South China Agricultural University Experimental Animal Welfare Ethics Committee and were performed in strict accordance with the approved guidelines.

### Viruses and Animals

The current epidemic H9N2 AIV (A/chicken/ Hunan/HN/2015) belonged to the h9.4.2.5 lineage, as described previously (34). The IBV belonged to the QX-like genotype, TJ401, as described previously (35). Both viruses were propagated in 10-day-old SPF chicken embryos and stored at −80°C for further analysis. The embryo 50% infectious doses (EID_50_) of these viruses were determined as described previously. One-day-old SPF chickens were purchased from Guangdong Wens Dahuanong Biotechnology Co., Ltd (production license: SCXK [Yue] 2018–0019) and housed in isolator cages.

### Animal Experiments

One-day-old SPF chickens were raised in isolators until they were 5 days old. Ninety-five 5-day-old SPF chickens were randomly divided into five groups, namely, the H9N2, IBV, IBV/H9N2, IBV+H9N2, and H9N2/IBV groups. Chickens in the H9N2 and IBV groups were inoculated intranasally with 10^6^ EID_50_ H9N2 virus and IBV, respectively (Table 1). In the IBV/H9N2 group, chickens received 10^6^ EID_50_ IBV, followed by 10^6^ EID_50_ H9N2 virus 2 days later. Chickens in the IBV+H9N2 group simultaneously received 10^6^ EID_50_ H9N2 virus and IBV. In the H9N2/IBV group, chickens were inoculated with 10^6^ EID_50_ H9N2, followed by 10^6^ EID_50_ IBV 2 days later. Ten chickens received phosphate-buffered saline (PBS) as the control group. Oropharyngeal and cloacal swabs were collected from each chicken and suspended in 1000 μL PBS at 3, 5, 7, 9, 11, and 13 dpi. At 3, 5, and 7 dpi, three chickens in each group were euthanized. Gross lesions were recorded as described previously (36), and the tracheal, lungs, and kidneys samples were collected for viral load testing using one-step quantitative reverse transcription polymerase chain reaction (RT-qPCR). All remaining chickens were monitored daily for mortality and morbidity for 14 days.

**Table 1:** Animal experimental design.

**Group**	**Number**	**Challenge virus**	
		Day 0	Day 2
H9N2	19	H9N2	PBS
IBV	19	IBV	PBS
IBV/H9N2	19	IBV	H9N2
IBV+H9N2	19	IBV+H9N2	PBS
H9N2/IBV	19	H9N2	IBV
Control	10	PBS	PBS

### Histopathology

The tracheal and kidney samples collected as described above were fixed in 10% neutral formalin for 48 h at room temperature. After fixation, the samples were embedded in paraffin, cut into 5 μm sections, and stained with modified hematoxylin and eosin. Microscopic lesions were examined using light microscopy.

### RNA Isolation, CDNA Synthesis, and RT-QPCR

Total RNA was isolated from the tissue and swab samples using the Total RNA Kit II (Omega Bio-Tek, Norcross, GA, USA) and RNAfast200 (Feijie, Shanghai, China), respectively, according to the manufacturer's instructions. Viral loads of H9N2 AIV and IBV in the tissues and swabs samples were detected using the HiScript II One Step qRT-PCR SYBR Green Kit (Vazyme, Nanjing, China). To investigate the mRNA expression of inflammatory cytokines, RNA from tested tissues samples was reverse-transcribed to cDNA using the HiScript II Q RT SuperMix for qPCR + gDNA wiper (Vazyme) and determined with qRT-PCR using SYBR® Premix Ex Taq™ (Takara). The primers are shown in Supplementary Table 1. Data analyses were performed using the 2^−ΔΔCt^ method.

To calculate the viral load, we generated a standard curve for each virus, as described previously. Briefly, the same region of the N gene of IBV and M gene of H9N2 virus was amplified and cloned into the pMD-19T vector (Takara) as the standard plasmid. We plotted the copy number of the standard plasmid for each dilution against the cycle threshold (CT) to generate a standard curve.

### Western Blot and Enzyme-Linked Immunosorbent Assay (ELISA) Analysis

The expression of NLPR3 protein in the trachea, lungs, and kidneys was detected using western blot, as described previously. The rabbit anti-NLRP3 antibody was provided by Professor Zhangyong Ning (South China Agricultural University). The expression of IL-1β in the trachea, lungs, and kidneys was also detected using a chicken IL-1β ELISA kit (USCN Sciences Co., Ltd., Wuhan, China).

### Transcriptome Analysis

Twenty 5-day-old SPF chickens were randomly divided into four groups, namely, the H9N2, IBV, IBV/H9N2, and control groups, and the operation of the viral-infection method for each group was conducted as described in 4.3. At 5 dpi, three chickens in each group were euthanized, and tracheal samples were taken and sent to Health Time Gene Co., Ltd. (Shenzhen, China), for transcriptome analysis as described previously (37).

### Statistical Methods

All data were analyzed using two-way analysis of variance with GraphPad Prism8.0 software. The data were expressed as means ± standard deviations, and a *P* < 0.05 was considered statistically significant. The “a” was used to indicated a significant difference (*P* < 0.05) between the three co-infection groups and the IBV group, while “b” was used to indicated a significant difference (*P* < 0.05) between the three co-infection groups and the H9N2 group.

## Results

### Clinical Signs and Mortality Rate

**Figure 1:**
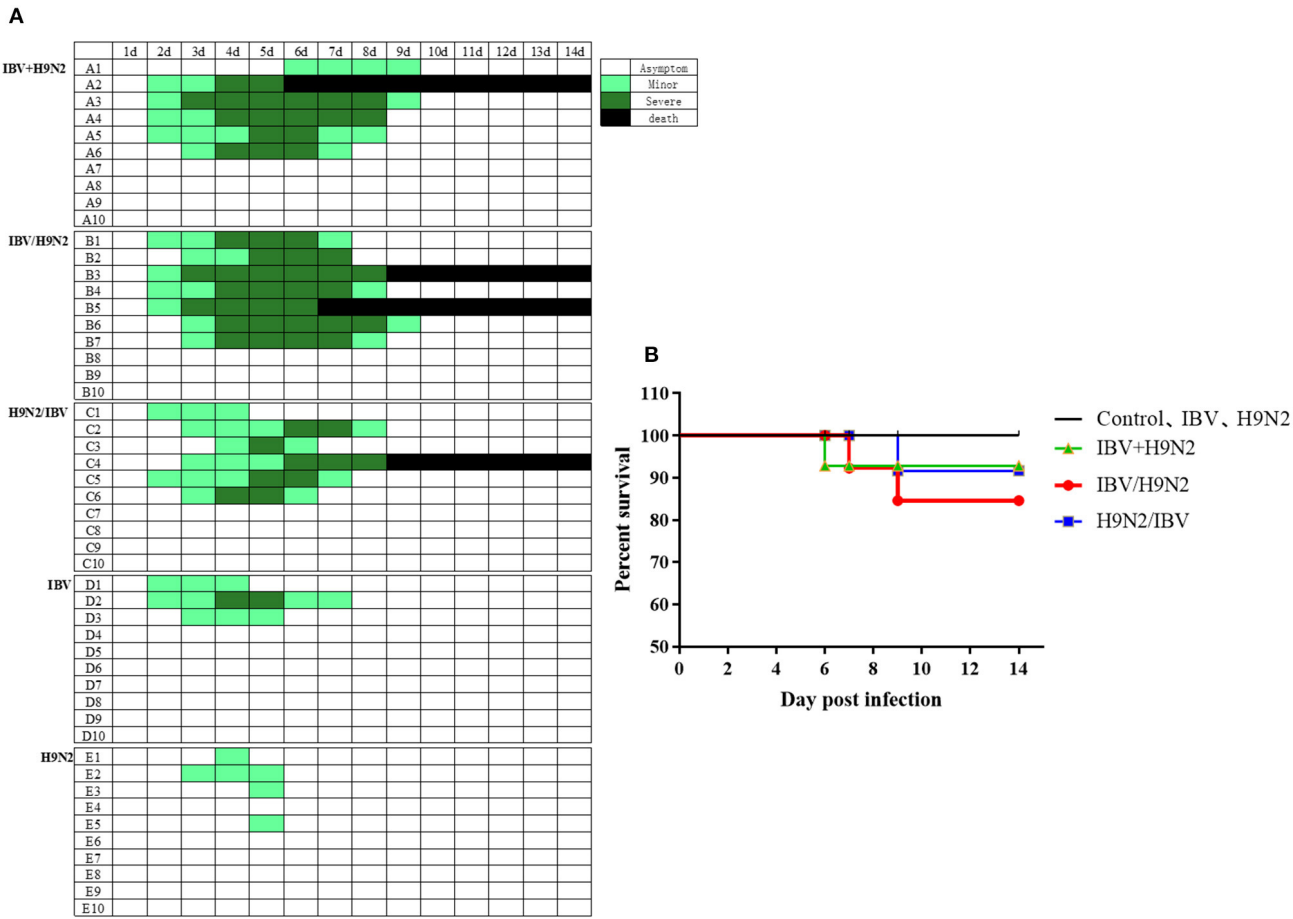
Clinical signs and mortality of chickens after co-infection with IBV and H9N2 virus. **(A)** Clinical symptoms were observed once daily, and scored according to degree of severity. **(B)** Mortality rates of chickens after co-infection with IBV and H9N2 virus. IBV, infectious bronchitis virus.

To evaluate the clinical signs caused by co-infection with IBV and H9N2 AIV in chickens, four groups of 19 one-day-old chickens were inoculated intranasally with the H9N2 virus, IBV, or both. The morbidity was 40 and 30% in the single-infection H9N2 and IBV groups, respectively. However, the morbidity was 60, 60, and 70% in the IBV+H9N2, H9N2/IBV, and IBV/H9N2 groups, respectively (Figure 1A). Only mild clinical signs such as sneezing, coughing, and head shaking were observed in the H9N2 and IBV groups, while severe respiratory signs such as rales and mouth breathing were recorded in the three co-infection groups. Furthermore, 20 (2/10), 10 (1/10), and 10% (1/10) of infected chickens died in the IBV/H9N2, IBV+H9N2, and H9N2/IBV groups, respectively, during the experiment (Figure 1B). None of the chickens in the H9N2 and IBV groups died during the observation period. Overall, co-infection with IBV and H9N2 AIV exhibited high morbidity and mortality.

### Effect of Co-infection With IBV and H9N2 Virus on the Kidneys

**Figure 2:**
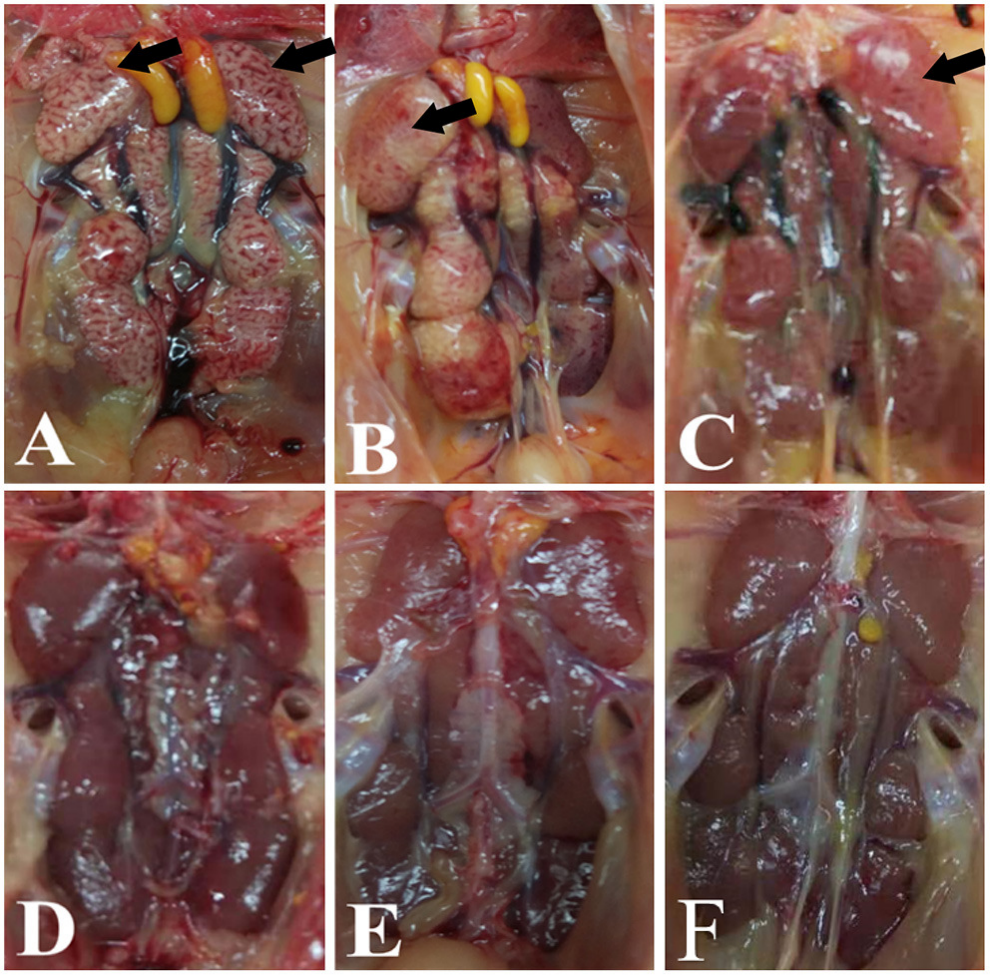
Gross lesions in the kidneys. **(A)** IBV/H9N2 group, **(B)** IBV+H9N2 group, **(C)** H9N2/IBV group, **(D)** IBV group, **(E)** H9N2 group, and **(F)** control group. Black arrows point to the signs of a “spotted kidney”. IBV, infectious bronchitis virus.

Significant swelling of the kidneys and signs of a “spotted kidney” were noted at necropsy in the IBV+H9N2, IBV/H9N2, and H9N2/IBV groups but not in the IBV and H9N2 group, while kidney swelling was observed in the IBV group (Figure 2). Notably, co-infection with IBV and H9N2 virus produced severe necropsy lesions in the kidneys of chickens, when compared with a single infection with IBV.

### Histopathological Damage in the Tracheas and Kidneys With Co-infection

**Figure 3:**
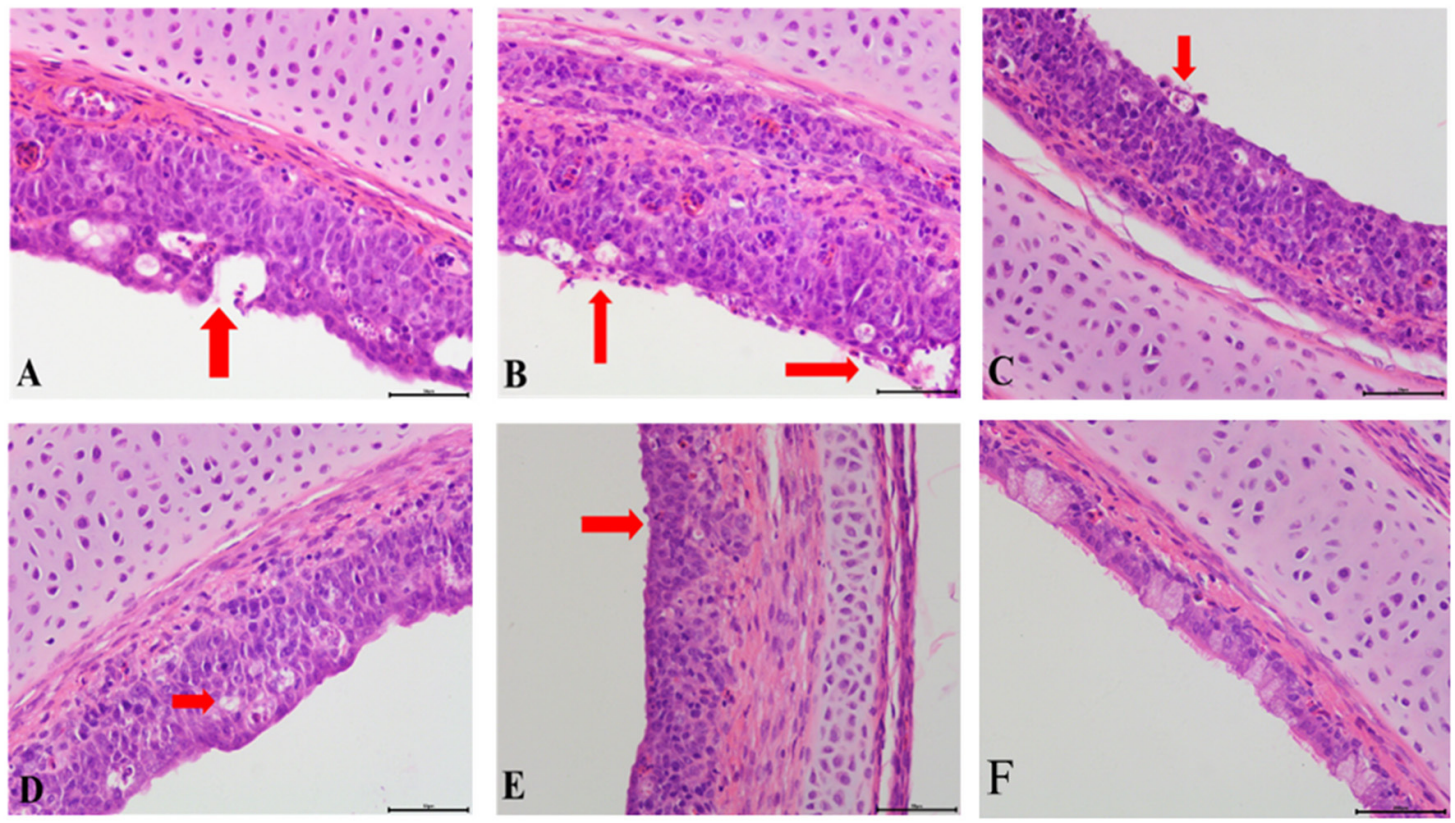
Tracheal histopathology at 5 dpi. **(A)** IBV/H9N2 group, **(B)** IBV+H9N2 group, **(C)** H9N2/IBV group, **(D)** IBV group, **(E)** H9N2 group, and **(F)** Control group. Red arrows point to the lesion. Magnification 400× , scalebar 50 μM. IBV, infectious bronchitis virus.

**Figure 4:**
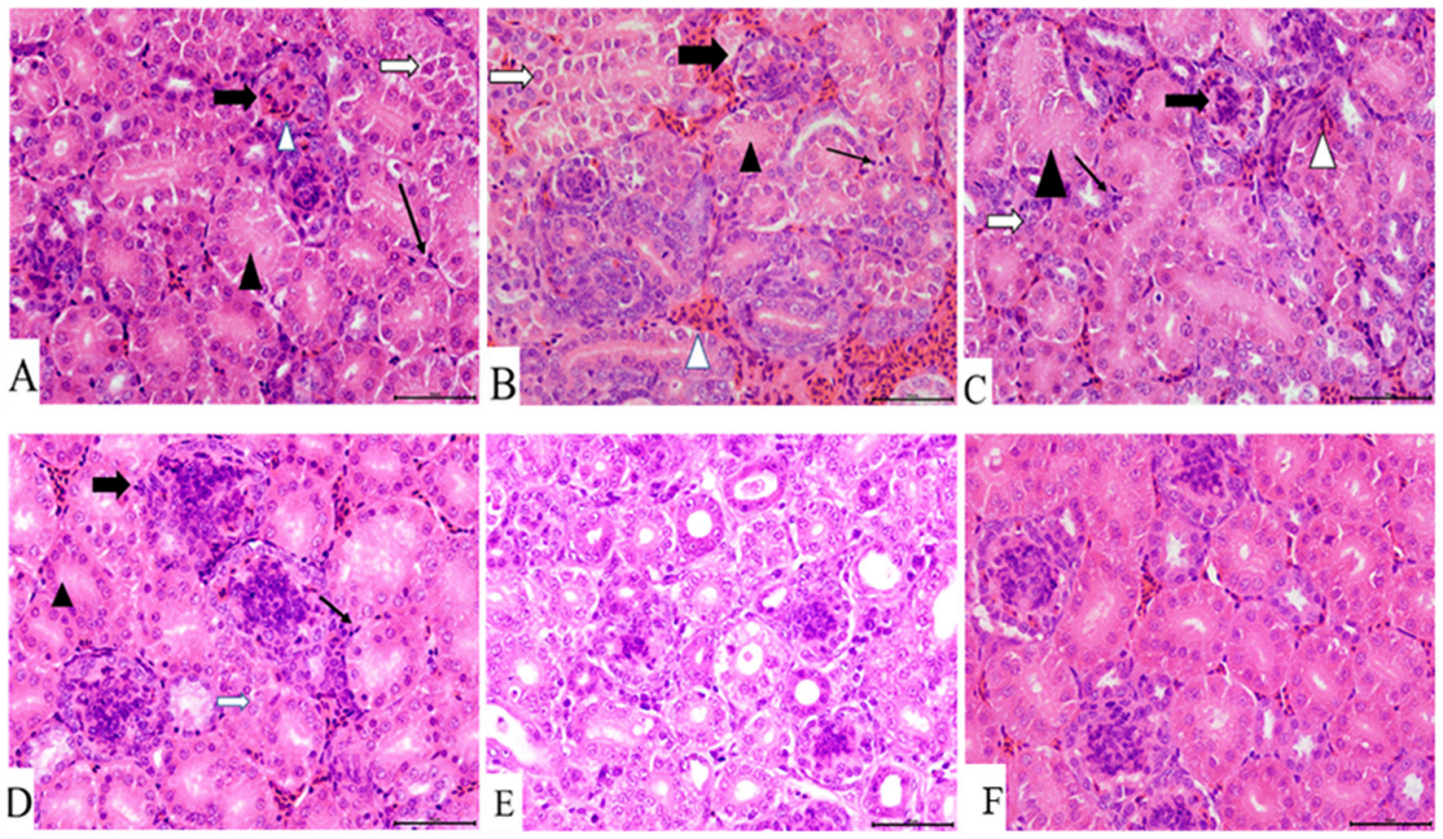
Kidney histopathology at 7 dpi. **(A)** IBV/H9N2 group, **(B)** IBV+H9N2 group, **(C)** H9N2/IBV group, **(D)** IBV group, **(E)** H9N2 group, and **(F)** control group. Black triangles donate absence of the renal tubular lumen. White arrows donate degeneration of the renal tubular epithelial cells. Black lined arrows point to the exudation of monocytes. Solid black arrows represent the glomerulus enlargement and the absence of the renal capsule cavity. Magnification 400×, scalebar 50 μM. IBV, infectious bronchitis virus.

Compared with the control group, obvious pathological changes in the trachea and kidneys in the co-infection groups were noted, especially in the IBV/H9N2 group. Varying degrees of epithelial degeneration of ciliated cells, hemorrhage, mucosal injury, and mononuclear cell infiltration could be observed in the tracheas of chickens in the co-infection groups (Figure 3). Similarly, interstitial nephritis, inflammatory cell infiltration, and hemorrhage were observed in the kidneys of chickens in the co-infection groups, especially in the IBV/H9N2 group (Figure 4). Only low levels of inflammatory cell infiltration were observed in the tracheas and kidneys of chickens in the IBV group and in the tracheas of chickens in the H9N2 group. Overall, co-infection with IBV and H9N2 virus could contribute to severe pathological damage due to the induced inflammation response.

### Viral Load and Viral Shedding

**Figure 5:**
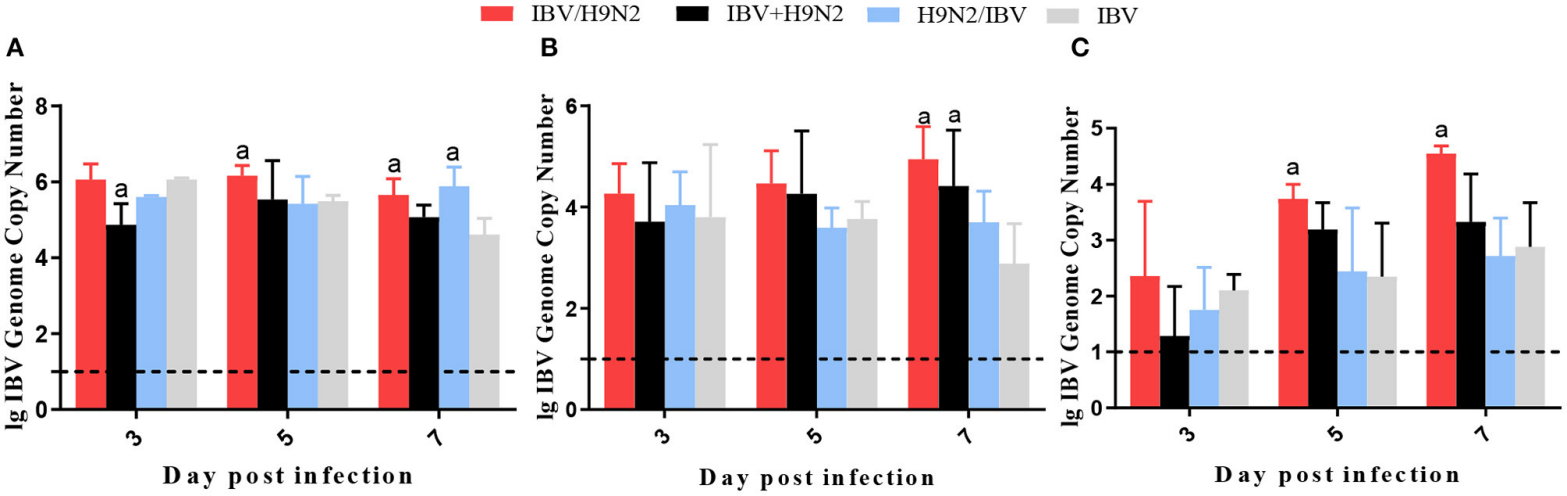
Viral load of IBV in chickens. To compare viral replication, certain tissues, including samples from the trachea, lungs, and kidneys, were collected at 3, 5, and 7 dpi for virus titration using one-step RT-qPCR. **(A)** Trachea, **(B)** lungs, and **(C)** kidneys. Data are presented as means ± SD. “a” indicates a significant difference (*P* < 0.05) between the three co-infection groups and the single-infection IBV group. Dashed black lines indicate the lower limit of detection. IBV, infectious bronchitis virus; RT-qPCR, quantitative reverse transcription polymerase chain reaction; SD, standard deviation.

**Figure 6:**
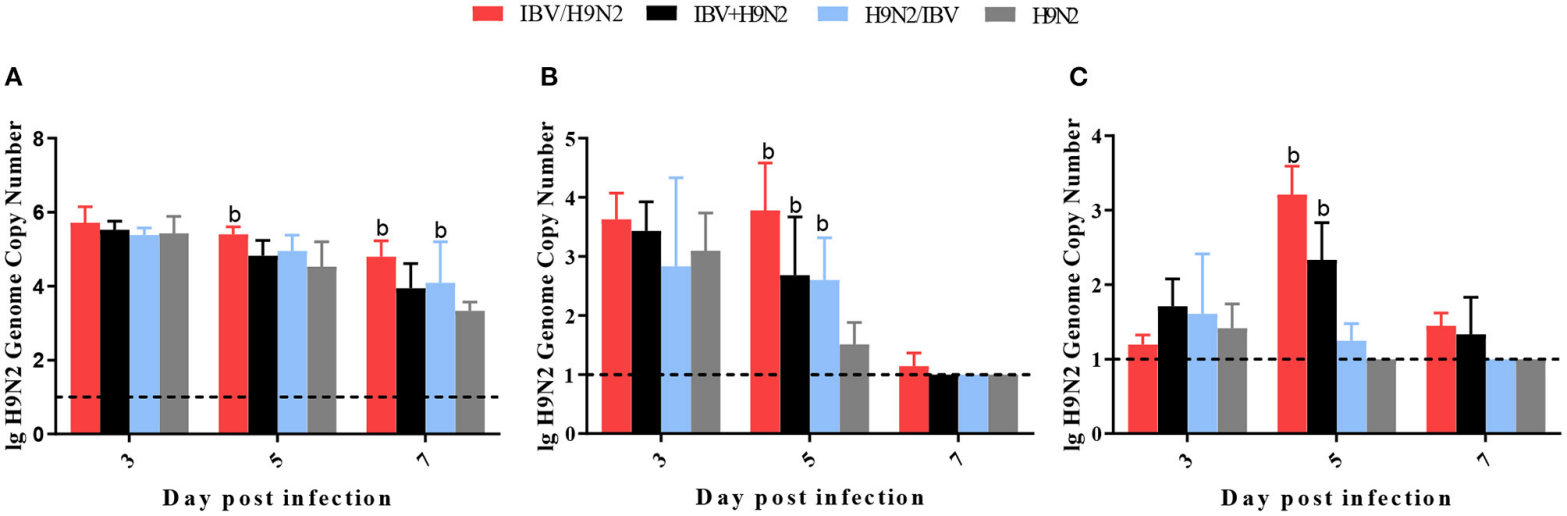
Viral load of H9N2 virus in chickens. To compare virus replication, certain tissues, including samples from the trachea, lungs, and kidneys, were collected at 3, 5, and 7 dpi for virus titration using one-step RT-qPCR. **(A)** Trachea, **(B)** lungs, and **(C)** kidneys. Data are presented as means ± SD. “b” indicates a significant difference (*P* < 0.05) between the three co-infection groups and the single-infection H9N2 group. Dashed black lines indicate the lower limit of detection. RT-qPCR, quantitative reverse transcription polymerase chain reaction; SD, standard deviation.

To compare virus replication in tissues, samples from the trachea, lungs, and kidneys were collected at 3, 5, and 7 days post infection (dpi) for virus titration. Compared with the single infection IBV group, the IBV loads in the trachea and kidneys were significantly higher (*P* < 0.05) in the IBV/H9N2 group at 5 and 7 dpi, as well as in the IBV+H9N2 group at 3 dpi and 7 dpi, respectively (Figure 5). Compared with the single-infection H9N2 group, the viral load of H9N2 virus demonstrated a significant upregulation (*P* < 0.05) in the following: the trachea at 5 and 7 dpi, and the lungs and kidneys at 5 dpi in the IBV/H9N2 group; the lungs and kidneys at 5 dpi, and the trachea at 7 dpi in the IBV+H9N2 group; and the lungs and kidneys at 5 dpi in the H9N2/IBV group (Figure 6).

**Figure 7:**
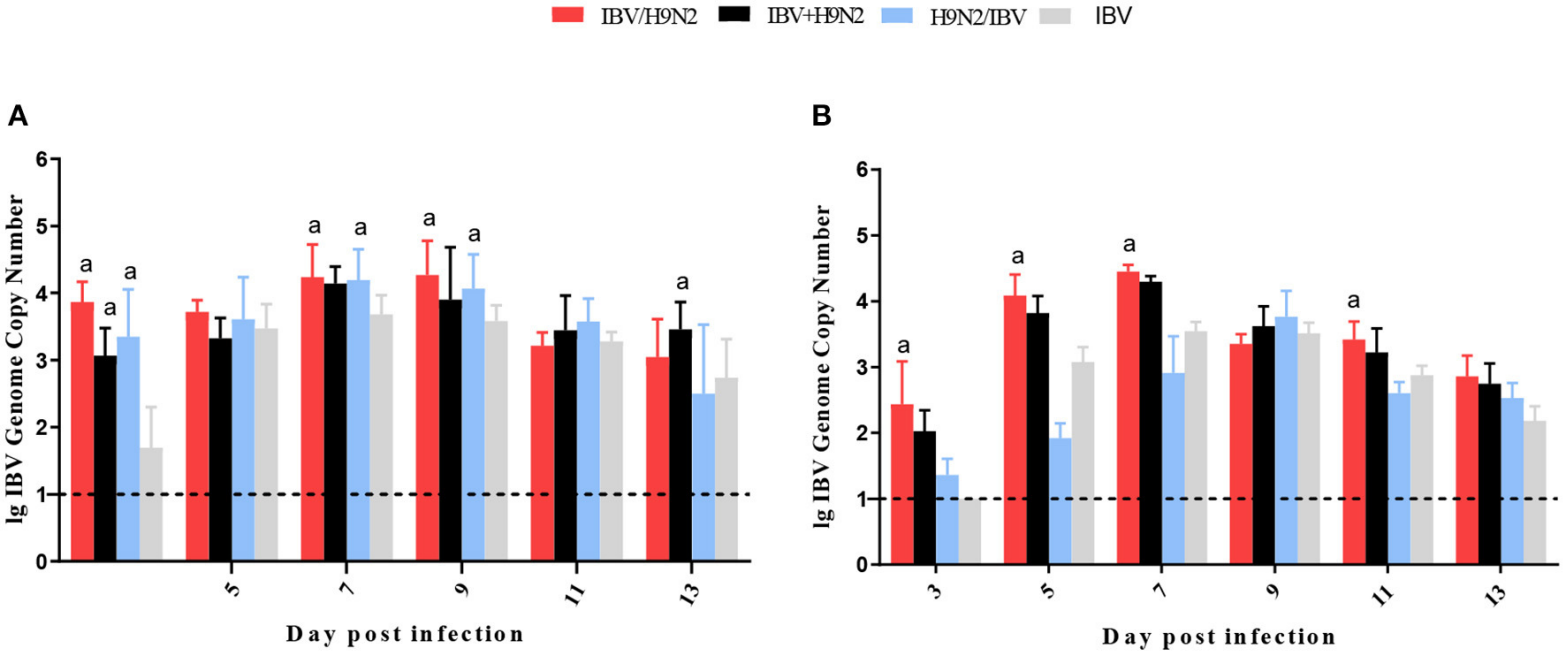
Viral shedding of IBV. To investigate the viral shedding of IBV virus, oropharyngeal **(A)** and cloacal **(B)** swabs were collected for virus detection at 3, 5, 7, 9, 11, and 13 dpi using one-step RT-qPCR. Data are presented as means ± SD. “a” indicates a significant difference (*P* < 0.05) between the three co-infection groups and the IBV group. Dashed black lines indicate the lower limit of detection. IBV, infectious bronchitis virus; RT-qPCR, quantitative reverse transcription polymerase chain reaction; SD, standard deviation.

**Figure 8:**
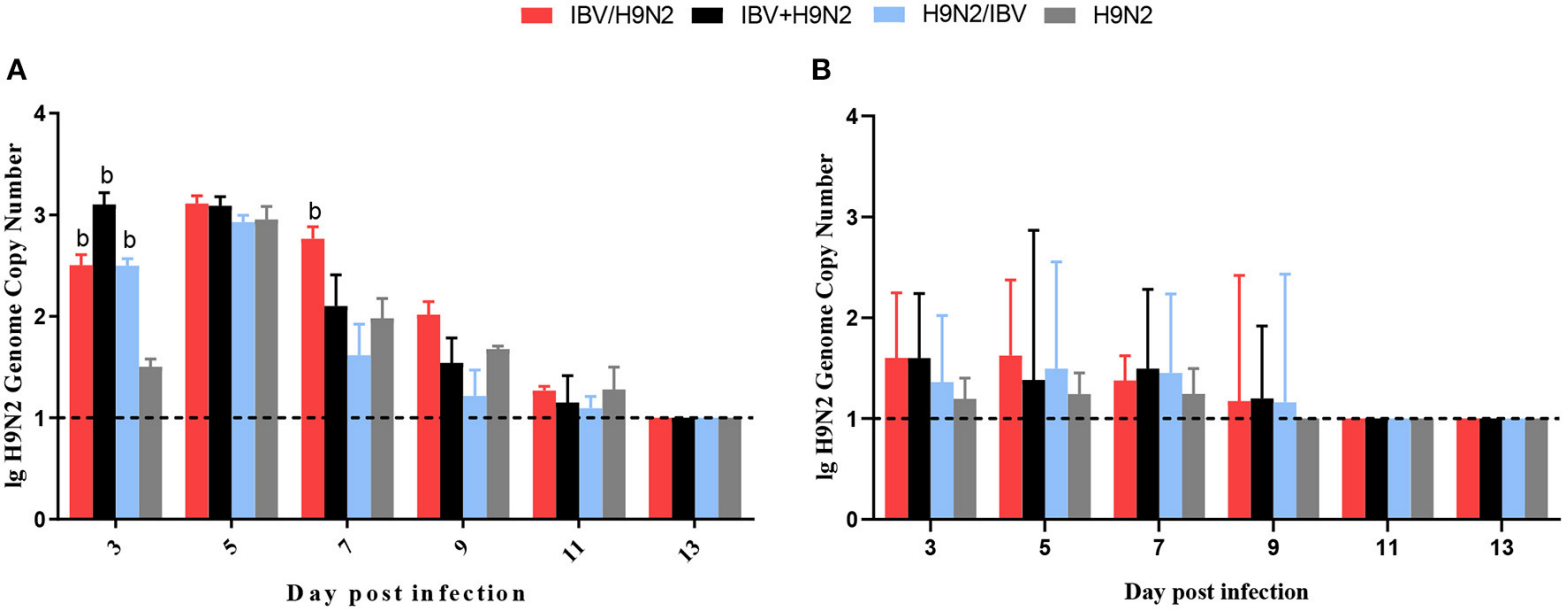
Viral shedding of H9N2 virus. To investigate the viral shedding of H9N2 virus, oropharyngeal **(A)** and cloacal **(B)** swabs were collected for virus detection at 3, 5, 7, 9, 11, and 13 dpi using one-step RT-qPCR. Data are presented as means ± SD. “b” indicates a significant difference (*P* < 0.05) between the three co-infection groups and the H9N2 group. Dashed black lines indicate the lower limit of detection. RT-qPCR, quantitative reverse transcription polymerase chain reaction; SD, standard deviation.

To investigate the viral shedding of IBV and H9N2 virus, oropharyngeal and cloacal swabs were collected for virus detection at 3, 5, 7, 9, 11, and 13 dpi. IBV shedding could be detected in the oropharyngeal and cloacal tissue in the single-infection IBV group and in the three co-infection groups on all testing days. The shedding titer of IBV in the oropharynx in the IBV/H9N2 group were significantly higher (*P* < 0.05) than those in the IBV group at 3, 7, and 9 dpi (Figure 7A). Significantly higher (*P* < 0.05) IBV shedding titers were also observed in the oropharyngeal region in the IBV+H9N2 group at 3 and 1 dpi, and in the IBV/H9N2 group at 7 and 9 dpi. Meanwhile, the shedding titers of IBV in the cloacal tissue in the IBV/H9N2 group were also significantly higher (*P* < 0.05) than those in the IBV group at 3, 5, 7, and 11 dpi (Figure 7B). The shedding titers of H9N2 virus via the oropharyngeal tissue in the three co-infection groups at 3 dpi and in the IBV/H9N2 group at 7 dpi were significantly higher (*P* < 0.05) than those in the H9N2 group (Figure 8A). For the cloacal samples, the shedding of H9N2 virus could be detected in the three co-infection groups and in the H9N2 group at 3, 5, 7, and 9 dpi, although the titers showed no significant difference between the co-infection and H9N2 groups (Figure 8B).

### Expression of Inflammatory Cytokines in the Trachea, Lungs, and Kidneys

**Figure 9:**
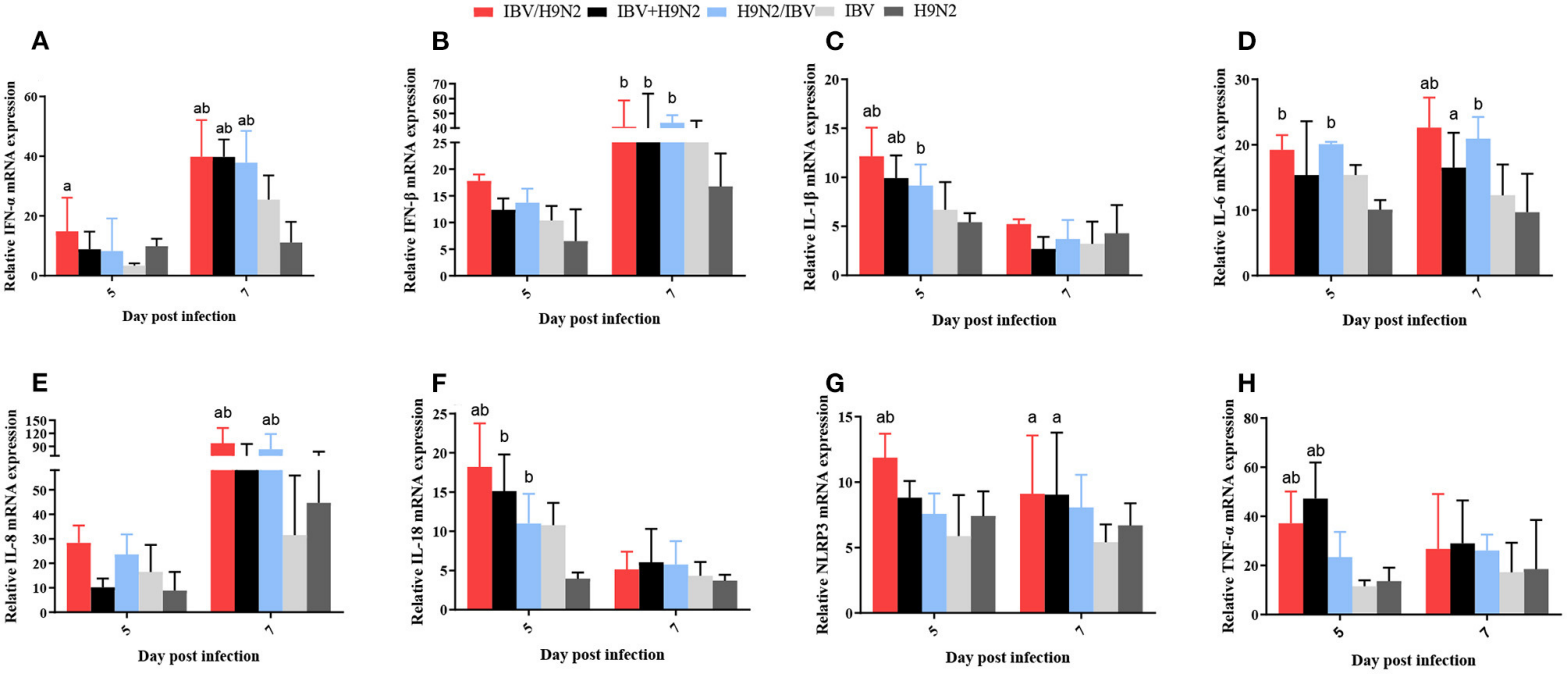
Effect of co-infection with IBV and H9N2 virus on inflammatory cytokines in the chicken trachea. **(A)** IFN-α, **(B)** IFN-β, **(C)** IL-1β, **(D)** IL-6, **(E)** IL-8, **(F)** IL-18, **(G)** NLRP3, and **(H)** TNF-α. Data are presented as means ± SD. “a” indicates a significant difference (*P* < 0.05) between the three co-infection groups and the IBV group. “b” indicates a significant difference (*P* < 0.05) between the three co-infection groups and the H9N2 group. “ab” indicates a significant difference (*P* < 0.05) between the three co-infection groups and the H9N2 group. IBV, infectious bronchitis virus; IFN, interferon; IL, interleukin; NLRP3, NLR family pyrin domain containing 3; TNF-α, tumor necrosis factor-α; SD, standard deviation.

Previous studies have demonstrated that the inflammatory response plays an important role in viral infection. In this study, we investigated the expression of inflammatory cytokines, including interleukin-1β (IL-1β), IL-6, IL-8, IL-18, NLR family pyrin domain containing 3 (NLRP3), tumor necrosis factor-α (TNF-α), interferon-α (IFN-α), and IFN-β, in the trachea, lungs, and kidneys. In the trachea, the transcriptional expression levels of inflammatory cytokines at 5 dpi (including IL-1β, IL-18, NLRP3, and TNF-α) and at 7 dpi (including IFN-α and IL-6) were significantly higher (*P* < 0.05) in the IBV/H9N2 group than in the IBV and H9N2 groups (Figure 9). The expression levels of IFN-α at 7 dpi and IL-1β and TNF-α at 5 dpi were also significantly higher (*P* < 0.05) in the IBV+H9N2 group than in the single-infection IBV and H9N2 groups. The expression levels of IFN-β at 7 dpi and IL-18 at 5 dpi were significantly higher (*P* < 0.05) in the IBV+H9N2 group than in the H9N2 group; those of IL-6 and NLRP3 at 7 dpi were significantly higher (*P* < 0.05) in the IBV+H9N2 group than in the IBV group. The expression levels of IFN-α and IL-8 at 7 dpi were significantly higher (*P* < 0.05) in the H9N2/IBV group than in the IBV and H9N2 groups. The IFN-β, IL-1β, and IL-18 mRNA expression at 5 dpi and the IL-6 mRNA expression at 5 and 7 dpi were significantly upregulated (*P* < 0.05) in the H9N2/IBV group, compared with the H9N2 group.

**Figure 10:**
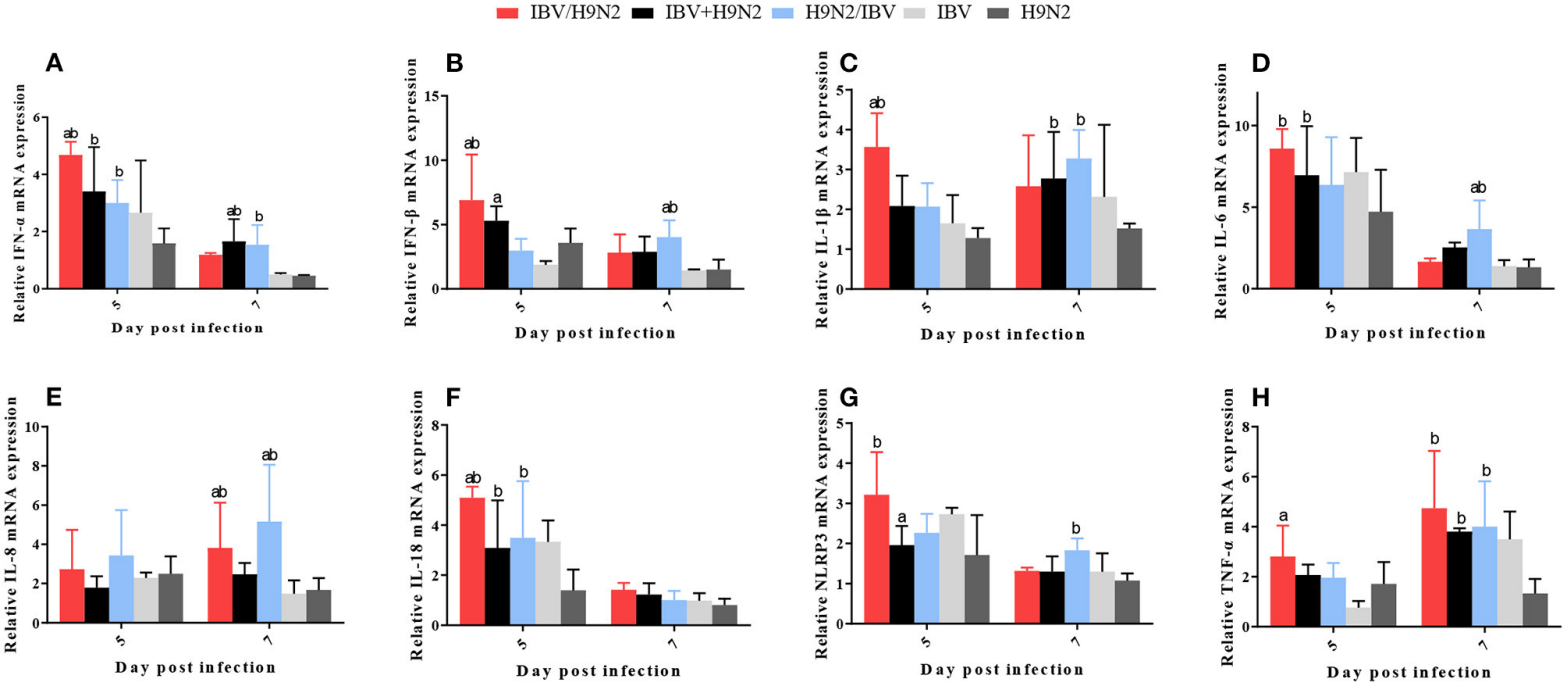
Effect of co-infection with IBV and H9N2 virus on inflammatory cytokines in the lungs. **(A)** IFN-α, **(B)** IFN-β, **(C)** IL-1β, **(D)** IL-6, **(E)** IL-8, **(F)** IL-18, **(G)** NLRP3, and **(H)** TNF-α. Data are presented as means ± SD. “a” indicates a significant difference (*P* < 0.05) between the three co-infection groups and the IBV group. “b” indicates a significant difference (*P* < 0.05) between the three co-infection groups and the H9N2 group. “ab” indicates a significant difference (*P* < 0.05) between the three co-infection groups and the H9N2 group. IBV, infectious bronchitis virus; IFN, interferon; IL, interleukin; NLRP3, NLR family pyrin domain containing 3; TNF-α, tumor necrosis factor-α; SD, standard deviation.

In the lungs, the expression levels of IFN-α, IFN-β, and IL-18 at 5 dpi and IL-8 at 7 dpi were significantly higher (*P* < 0.05) in the IBV/H9N2 group than in the IBV and H9N2 groups (Figure 10). The IL-6 and NLRP3 mRNA expression at 5 dpi and the TNF-α mRNA expression at 7 dpi showed significant upregulation (*P* < 0.05) in the IBV/H9N2 group, compared with the H9N2 group. The expression of IL-6 and IL-18 at 5 dpi, IL-1β and TNF-α at 7 dpi, as well as IFN-α at 5 and 7 dpi were significantly higher (*P* < 0.05) in the IBV+H9N2 group than in the H9N2 group; the expression levels of IFN-α at 5 and 7 dpi, IL-18 at 5 dpi, as well as IL-1β, NLRP3, and TNF-α at 7 dpi were also significantly higher (*P* < 0.05) in the H9N2 /IBV group. The expression levels of IFN-β, IL-6, and IL-8 at 7 dpi were also significantly higher (*P* < 0.05) than in the IBV and H9N2 groups.

**Figure 11:**
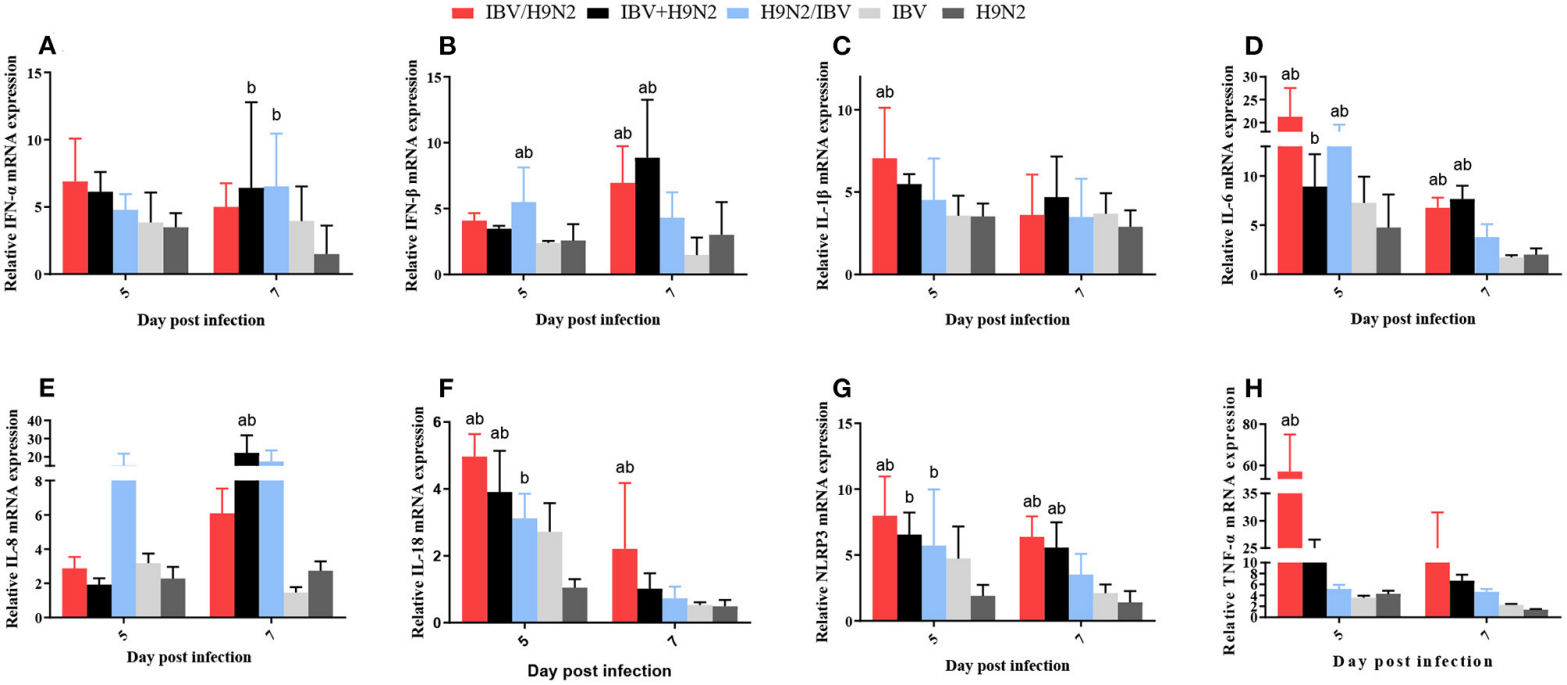
Effect of co-infection with IBV and H9N2 virus on inflammatory cytokines in the kidneys. **(A)** IFN-α, **(B)** IFN-β, **(C)** IL-1β, **(D)** IL-6, **(E)** IL-8, **(F)** IL-18, **(G)** NLRP3, and **(H)** TNF-α. Data are presented as means ± SD. “a” indicates a significant difference (*P* < 0.05) between the three co-infection groups and the IBV group. “b” indicates a significant difference *(P* < 0.05) between the three co-infection groups and the H9N2 group. “ab” indicates a significant difference (*P* < 0.05) between the three co-infection groups and the H9N2 group. IBV, infectious bronchitis virus; IFN, interferon; IL, interleukin; NLRP3, NLR family pyrin domain containing 3; TNF-α, tumor necrosis factor-α; SD, standard deviation.

In the kidneys, the expression levels of IL-1β and TNF-α at 5 dpi, IFN-β at 7 dpi, and IL-6, IL-18, and NLRP3 at 5 and 7 dpi were significantly higher (*P* < 0.05) in the IBV/H9N2 group than in the IBV and H9N2 groups (Figure 11). Compared with the IBV and H9N2 groups, the expression levels of IL-18 at 5 dpi, IFN-β and IL-8 at 7 dpi, and NLRP3 at 5 and 7 dpi were significantly upregulated in the IBV+H9N2 group, and the expression levels of IFN-β and IL-6 at 5 dpi were also significantly upregulated in the H9N2/IBV group (*P* < 0.05). Overall, these results indicated that co-infection with IBV and H9N2 virus could induce a severe inflammatory response in the tissues of chickens.

**Figure 12:**
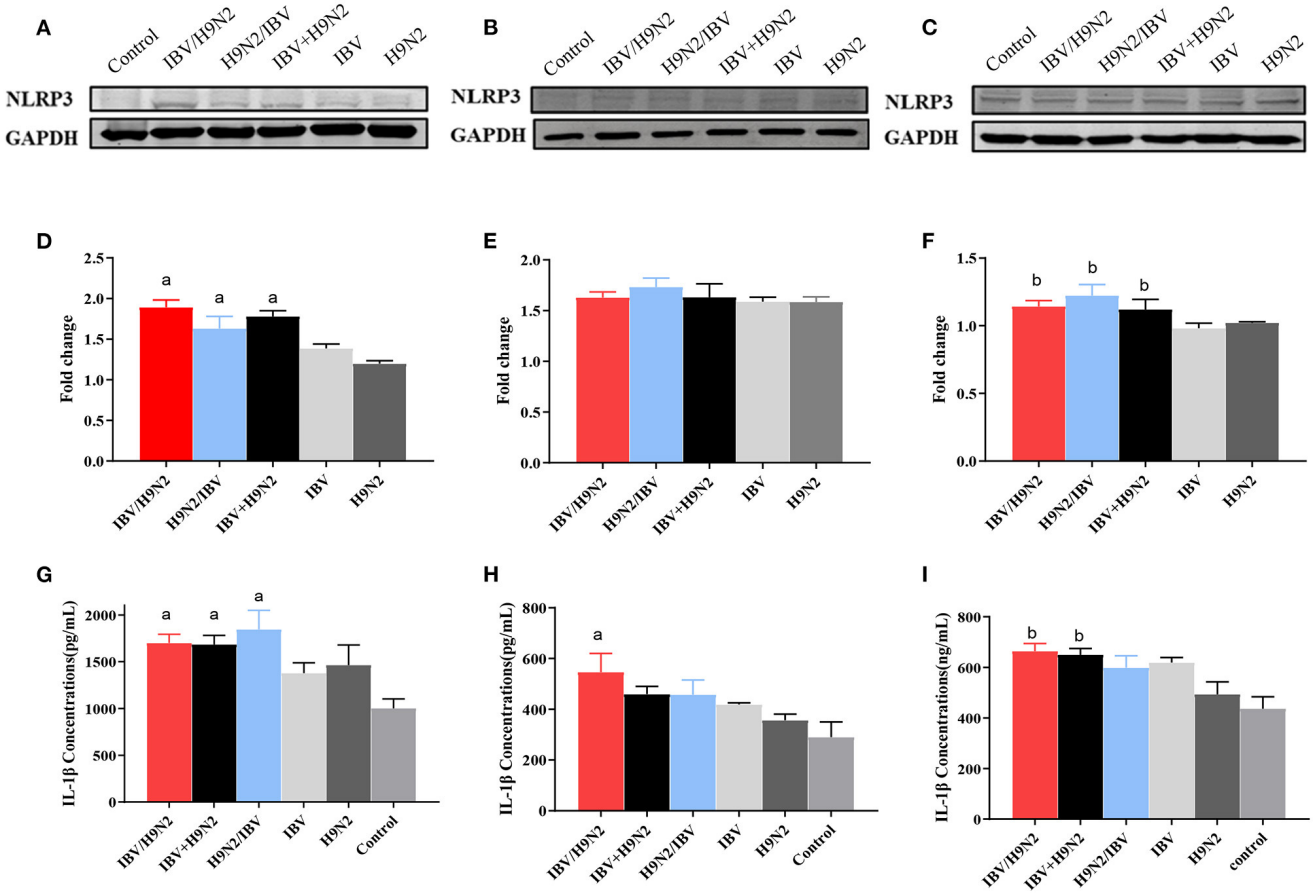
Effect of co-infection with IBV and H9N2 virus on the NLRP3 inflammasome. Samples from the trachea, lungs, and kidneys were collected at 5 dpi. The protein expression of NLRP3 in the trachea, lungs, and kidneys was determined using a western blot. The expression of mature IL-1β was determined using ELISA. Protein expression levels of NLRP3 in the trachea **(A)**, lungs **(B)**, and kidneys **(C)**. Intensity band ratios of NLRP3 to GAPDH in the trachea **(D)**, lungs **(E)**, and kidneys **(F)**. Expression levels of mature IL-1β in the trachea **(G)**, lungs **(H)**, and kidneys **(I)**. Data are presented as means ± SD. “a” indicates a significant difference (*P* < 0.05) between the three co-infection groups and the IBV group. “b” indicates a significant difference (*P* < 0.05) between the three co-infection groups and the H9N2 group. IBV, infectious bronchitis virus; NLRP3, NLR family pyrin domain containing 3; ELISA, enzyme-linked immunosorbent assay; GAPDH, glyceraldehyde 3-phosphate dehydrogenase; SD, standard deviation.

The NLRP3 inflammasome controls the maturation and secretion of IL-1β and IL-18 and plays an important role in the inflammatory response. We found that the protein expression of NLRP3 was significantly higher in the trachea and kidneys in the three co-infection groups than in the H9N2 and IBV groups (Figures 12A–F). Meanwhile, the level of IL-1β secretion also showed a similar tendency (Figures 12G–I). Overall, co-infection with IBV and H9N2 virus increased the activity of the NLRP3 inflammasome.

### Transcriptome Analysis of Trachea of SPF Chickens Co-infected With IBV and H9N2 AIV

**Figure 13:**
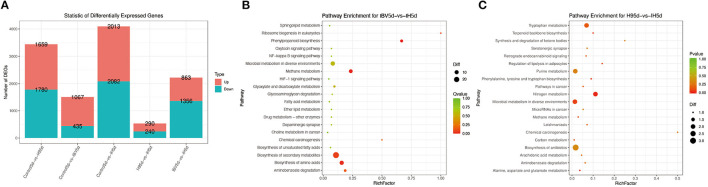
Results of transcriptome analysis. **(A)** Statistical graph of the number of DEGs between samples. Top 20 enriched pathways based on DEGs in IBV-vs-IBV/H9N2 **(B)** and H9N2-vs-IBV/H9N2 **(C)**. Rich factor is the ratio of the number of DEGs noted in the pathway terms to all gene numbers noted in this pathway term. A greater rich factor indicates higher intensiveness. *Q*-value is the corrected *P*-value ranging from 0 to 1 (green). A lower *Q*-value indicates higher intensiveness.

To investigate the immune mechanisms in SPF chickens co-infected with IBV and H9N2 AIV under different infection conditions, high-throughput transcriptome sequencing and differential analysis were performed on tracheal samples of chickens from the IBV, H9N2, IBV/H9N2, and control groups. By filtering low-quality data from the original sequencing data, the obtained data set showed that the sequencing data volume and data quality met the requirements of subsequent analysis (Supplementary Table 2). The differentially expressed genes (DEGs) in each comparison group were compared, and the number of DEGs in each comparison group was obtained through statistical analysis (Figure 13A, Supplementary Figure 1). Possible biological interactions of DEGs were detected by performing enrichment analysis of the GO and KEGG pathways. In the GO analysis, immune-related DEGs of H9N2-vs-IBV/H9N2 and IBV-vs-IBV/H9N2 were mainly involved in the immune response (GO: 0006955), inflammatory response (GO: 0006954), cytokine activity (GO: 0005125), and chemokine activity (GO: 0008009) (Supplementary Tables 3, 4). Furthermore, DEGs were mapped into the KEGG pathway database to further explain the individual function analysis; and the top 20 enriched pathways in H9N2-vs-IBV/H9N2 and IBV-vs-IBV/H9N2 are summarized in Figures 13B,C. Compared with the IBV group, the tryptophan metabolism, purine metabolism, nitrogen metabolism, and biosynthesis of antibiotics as signaling molecules were enriched in the IBV/H9N2 group. Meanwhile, compared with the H9N2 group, signaling pathways such as biosynthesis of secondary metabolites and nuclear factor κB (NF-κB) were enriched in the IBV/H9N2 group. Moreover, enrichment results of immune-related pathways were specifically extracted, which indicated enrichment of the NF-κB signaling pathway (Table 2). It is indicated that the cyclo-oxgen-ase 2 (COX2, genes list: 396451) was simultaneously enriched to NF-κB signaling pathway in IBV-vs-IBV/H9N2 and H9N2-vs-IBV/H9N2, which is evidence the previous results with both viruses can induce a stronger inflammatory response in chicken.

**Table 2:** Immune-related pathway enrichment statistics of DEGs upon in chicken trachea of different infection groups.

**Group**	**Pathway**	**Rich factor**	***P*** **-value**	**Q value**	**Pathway ID**	**Genes list**	**KOs**
IBV-vs-IBV/H9N2	NF-κB	0.0714	0.7095	0.9007	KO04064	396451	K11987
H9N2-vs-IBV/H9N2	NF-κB	0.0714	0.1423	0.2731	KO04064	396451	K11987

*DEGs, differentially expressed genes; KO, knockout; IBV, infectious bronchitis virus; NF-κB, nuclear factor κB*.

## Discussion

As the two most common respiratory pathogens, IBV and H9N2 AIV are widespread in poultry in China, resulting in heavy economic losses. However, these two viruses have usually shown low mortality rates under experimental conditions, especially for H9N2 AIV, inducing only mild respiratory symptoms, sometimes even without obvious clinical signs (10, 34, 38, 39). In poultry, mixed infections with multiple pathogens are very common. Mixed infections may increase the pathogenicity of pathogens and mortality rates of poultry (16, 40). There are also reports that IBV and H9N2 AIV interfere with each other after a mixed infection (41, 42). Mixed infection with a virus can affect viral shedding and the replication of the pathogen in the host and complicate the disease diagnosis (43, 44). It has been reported that IBV and H9N2 AIV can exist as a mixed infection, leading to increased pathogenicity (22, 45), indicating that there is an interaction between the two viruses. The sequential order of infection in mixed infections can lead to pathogenic differences (42, 46, 47), and compared with simultaneous infections, sequential infections more closely resemble natural infections. A recent study suggested that co-infection with IBV and H9N2 AIV might change the HA stability and function of H9N2, resulting the enhancement of the H9N2 pathogenicity (48). To investigate the role of IBV and H9N2 AIV in the respiratory disease of chickens in China, we systematically analyzed the pathogenicity of co-infection with IBV and H9N2 AIV circulating in China.

In this study, severe respiratory signs were observed in the IBV and H9N2 AIV co-infection groups, while only mild respiratory symptoms were observed in the H9N2 and IBV groups. Furthermore, co-infection with IBV and H9N2 AIV induced a 10–20% mortality rate in chickens; however, no chicken died in the single-infection IBV or H9N2 groups. The morbidity and mortality rates were 70 and 20% in the IBV/H9N2 group, respectively, which were higher than those in the IBV+H9N2 and H9N2/IBV groups. When compared with the IBV and H9N2 groups, the three co-infection groups had severe lesions in the kidneys, which were more severe in the IBV/H9N2 group than in the IBV+H9N2 and H9N2/IBV groups. The histopathological lesions in the trachea and kidneys also showed similar results. We found that co-infection with IBV and H9N2 virus could induce severe clinical outcomes, high morbidity, and high mortality in chickens, compared with a single infection with either virus, which is generally consistent with the results of previous studies (30, 31, 49, 50). Of note, co-infection with SARS-CoV-2 and A(H1N1) pdm09 virus could also induce more severe disease than a single infection with either virus in hamsters (51). These findings indicate that we should not ignore the presence of mixed infections during the diagnosis and treatment of patients with SARS-CoV-2 infection. Furthermore, we also showed that primary infection with IBV might play a major role in the development of respiratory disease, and secondary infection with H9N2 virus further enhances morbidity and mortality.

We also found that the viral loads (for IBV and H9N2 virus) were significantly higher in the tested tissues, including the trachea, lungs, and kidneys, in the co-infection groups than in the IBV or H9N2 group, indicating that co-infection promoted the propagation of IBV and H9N2 viruses. In a previous study, Haghighat-Jahromi et al. showed that the live IBV vaccine strain H120 could enhance the replication of H9N2 AIV (A/chicken/Iran/SH-110/99) (31). Some researchers supported the hypothesis that trypsin-like serine protease encoded by IBV could facilitate the cleavage activation of the hemagglutinin of H9N2 virus, increasing the replication of H9N2 virus (52, 53). However, Aouini et al. demonstrated that co-infection with IBV strain H120 and H9N2 AIV (A/Chicken/TUN/145/12) inhibited the replication of both viruses in primary chick embryo lung cells and SPF chicken eggs (32). The different results shown *in vivo* and *in vitro* could possibly be due to more complicated factors such as the immune response involved in infection *in vivo*. Thus, the mechanisms of enhanced replication of IBV and H9N2 virus in the co-infection groups need to be further investigated *in vivo*.

The pathogenicity of infectious microorganisms mainly depends on the replication capacity and host immune response, such as the inflammatory response. The inflammatory response is a double-edged sword: while fighting infection, it is also accompanied by tissue damage. If the immune system is activated increasingly without control, it will cause the expression of a large number of cytokines and aggregation of immune cells, thereby damaging the host tissues and increasing morbidity and mortality (54, 55). For example, in the case of severe acute respiratory syndrome coronavirus infection in 2004 and SARS-CoV-2 infection in 2020, the main reason for the high morbidity and mortality was the excessive cytokine response and massive recruitment of inflammatory lymphocytes caused by viral infection (56, 57). Previous studies have demonstrated that virulent IBV can induce excessive production of pro-inflammatory cytokines, which are associated with lesions in the trachea and kidneys (58, 59). Induction of inflammatory cytokines was also observed in chickens after infection with H9N2 AIV (60). In our study, the expression levels of inflammatory cytokines such as IFN-β, IL-1β, and TNF-α in the trachea, lungs, and kidneys were greater in the co-infection groups, especially in the IBV/H9N2 group, than in the single-infection IBV and H9N2 groups. Thus, the rapid replication of the virus in chickens can significantly increases the expression of inflammatory cytokines, related pattern recognition receptors, and inflammatory proteins. Excessive amounts of inflammatory factors and inflammatory proteins can cause a strong inflammatory response, resulting in more serious tissue damage.

The proinflammatory cytokine IL-1β is a key mediator of the inflammatory response (61). The maturation and secretion of proinflammatory cytokines, IL-1β and IL-18, require activation of the NLRP3 inflammasome (62, 63). The activation of the NLRP3 inflammasome requires two steps: the priming step, which is the upregulation of NLRP3 expression, and the activation step that assembles and activates the NLRP3 inflammasome (64, 65). Several RNA viruses, including Newcastle disease virus (66), influenza virus (67), severe acute respiratory syndrome coronavirus (68), and porcine reproductive and respiratory syndrome virus (69), have been reported to activate the NLRP3 inflammasome, leading to the secretion of IL-1β and IL-18. In the present study, we found that the expression levels of NLRP3 and IL-β were significantly higher in the trachea and kidneys of the co-infection groups, especially in the IBV/H9N2 group, than in the single-infection IBV and H9N2 groups. In the lungs, the protein expression of NLRP3 showed no significant difference among the infection groups, although the protein expression of IL-1β was slightly higher in the IBV group than in the H9N2 group. Meanwhile, the results of transcriptome analysis of the trachea of SPF chickens showed that co-infection with IBV and H9N2 virus could induce a stronger immune response, especially an inflammatory response. These results differ from the report in which the expression of IL-1β was inhibited after co-infection with H9N2 virus and IBV in embryo lung cells, especially simultaneous infection with both viruses (32). A previous study showed that the H7N9 AIV could induce lethal inflammation in mammals via activation of the NLRP3 inflammasome (70). These findings indicate that co-infection with IBV and H9N2 virus could increase the activation of NLRP3 inflammasome and exacerbate the inflammatory response, resulting in tissue lesions.

## Conclusion

We found that co-infection with IBV and H9N2 AIV could induce worse clinical outcomes and high morbidity and mortality in chickens. Furthermore, primary infection with IBV might play a major role in the development of respiratory disease in chickens, and secondary infection with H9N2 virus might further enhance pathogenicity. Co-infection with IBV and H9N2 virus might increase the pathogenicity by inducing a strong inflammatory response. Therefore, these findings suggest that the co-infection with IBV and H9N2 deserves more attention and the threat should not be ignored. Moreover, we lay a foundation for the further study of the synergistic infection mechanism of IBV and H9N2 AIV and provide a scientific basis for the prevention of mixed infections by these viruses in clinical practice.

## Data Availability Statement

The datasets presented in this study can be found in online repositories. The names of the repository/repositories and accession number(s) can be found in the article/Supplementary Material.

## Ethics Statement

The animal study was reviewed and approved by South China Agricultural University Experimental Animal Welfare Ethics Committee.

## Author Contributions

LK, RY, LC, and TR designed the experiments. LK, DZ, RY, and BX performed the experiments. LC, QY, JL, and QL assisted with the animal experiments. LK and RY drafted the manuscript. LC, TR, ML, and CD reviewed and revised the manuscript. All authors have read and approved the final manuscript.

## Funding

This research was funded by the National Key Research and Development Program of China (2018YFD0500100) and the Scientific and Technological Research Project of Foshan (2020001000151).

## Conflict of Interest

The authors declare that the research was conducted in the absence of any commercial or financial relationships that could be construed as a potential conflict of interest.

## Publisher's Note

All claims expressed in this article are solely those of the authors and do not necessarily represent those of their affiliated organizations, or those of the publisher, the editors and the reviewers. Any product that may be evaluated in this article, or claim that may be made by its manufacturer, is not guaranteed or endorsed by the publisher.
