# Supplementary material for: Infectious Bronchitis Virus Infection Increases Pathogenicity of H9N2 Avian Influenza Virus by Inducing Severe Inflammatory Response
Source: Front Vet Sci. 2022 Feb 8;8:824179. doi: 10.3389/fvets.2021.824179 (PMC8860976; doi:10.3389/fvets.2021.824179)
Supplement: Supplementary file 1 [file Data_Sheet_1.DOCX]

Supplementary Material

# Supplementary Tables

Table S1. Primers used in this study

| **Gene** | **Primer** | **Sequence (5′–3′)** | **Gen Bank Accession No.** |
| --- | --- | --- | --- |
| *IL-18* | Forward | CAGTTGCTTGTGGTTCGTCC | AB559577.1 |
|  | Reverse | CGCGGTGGTTTTGTAACAGT |  |
| *NLRP3* | Forward | CACACAAACACTCCTTGAACCA | HQ730914.1 |
|  | Reverse | GTCCCTTCCACCCACTCCATCAT |  |
| *TNFα* | Forward | CCGCCCAGTTCAGATGAGTT | NM_204267.1 |
|  | Reverse | GCAACAACCAGCTATGCACC |  |
| *IL-1β* | Forward | AACCCGACCAGGTCAACA | AJ245728.1 |
|  | Reverse | CGGTACATACGAGATGGAAAC |  |
| *IL-6* | Forward | ATCCCTCCTCGCCAATCTG | HM179640.1 |
|  | Reverse | CCTCACGGTCTTCTCCATA |  |
| *IFN-α* | Forward | GGACATGGCTCCCACACTAC | X92476.1 |
|  | Reverse | TCCAGGATGGTGTCGTTGAAG |  |
| *IFN-β* | Forward | TTCTCCTGCAACCATCTTC | AY974089.1 |
|  | Reverse | GAGGTGGAGCCGTATTCT |  |
| *IL-8* | Forward | CACAGCTCCACAAAACCTCA | NM_205498 |
|  | Reverse | GTCCTACCTTGCGACAGAGC |  |
| *GAPDH* | Forward | GGTGGTGCTAAGCGTGTTA | X01578.1 |
|  | Reverse | CCCTCCACAATGCCAA |  |
| *IBV* | Forward | CTGCCAAGGGTGCTGATGTAA | /^a^ |
|  | Reverse | CTTCCACTCCTACCACGATTCA |  |
| *H9N2* | Forward | TTACTCAACTGGTGCGCTTG | /^b^ |
|  | Reverse | GATGTTGTGCGTCAGCAATC |  |

^a^ Primers were designed based on the N gene of the IBV strain, TJ401.

^b^ Primers were designed based on the M gene of the H9N2 AIV strain (A/chicken/ Hunan/HN/2015).

Table S2 Data quality control table

| Samples | Total Raw Bases (G) | Total Clean Bases (G) | Total Clean Bases Ratio (%) | Clean Reads Q30 (%) |
| --- | --- | --- | --- | --- |
| H9-5d-1 | 7.21 | 7.21 | 99.99 | 94.77 |
| H9-5d-2 | 5.73 | 5.73 | 100 | 95.54 |
| H9-5d-3 | 5.82 | 5.82 | 100 | 94.97 |
| IBV-5d-1 | 6.62 | 6.62 | 100 | 95.06 |
| IBV-5d-2 | 5.42 | 5.39 | 99.43 | 95.57 |
| IBV-5d-3 | 6.48 | 6.47 | 100 | 95.31 |
| IH-5d-1 | 6.85 | 6.76 | 98.68 | 95.19 |
| IH-5d-2 | 6.10 | 6.05 | 99.25 | 95.05 |
| IH-5d-3 | 5.65 | 5.65 | 100 | 95.58 |
| Control-5d-1 | 6.85 | 6.76 | 98.68 | 95.3 |
| Control-5d-2 | 7.07 | 7.03 | 99.43 | 94.86 |
| Control-5d-3 | 7.35 | 7.35 | 99.99 | 94.84 |

Table S3. Immune-related GO enrichment statistics of H9N2-vs-IBV/H9N2 DEGs

| Class | GO ID | Term | DEGs in Term | All Gene in Term | P-value | Correct P-Value | DEGs list |
| --- | --- | --- | --- | --- | --- | --- | --- |
| BP | GO:0006955 | immune response | 16 | 103 | 7.82E-10 | 1.27E-06 | 417536;106182184;422511;396495;100858035;395196;101747454;395872;396216;395908;422654;100857191;101752166;417464;395082;428264 |
| MF | GO:0008009 | chemokine activity | 8 | 24 | 2.56E-08 | 3.65E-05 | 417536;422511;396495;395872;422654;100857191;417464;395082 |
| MF | GO:0005125 | cytokine activity | 7 | 18 | 5.70E-08 | 4.06E-05 | 100859040;395196;396216;101752166;101751261;428264;428665 |
| BP | GO:0006954 | inflammatory response | 4 | 27 | 0.00260538 | 0.3729792 | 100859040;395196;396451;428665 |
| BP | GO:0050727 | regulation of inflammatory response | 2 | 5 | 0.00451613 | 0.52516156 | 101747386;426356 |
| MF | GO:0016494 | C-X-C chemokine receptor activity | 1 | 2 | 0.04300654 | 1 | 430652 |
| BP | GO:0019882 | antigen processing and presentation | 2 | 10 | 0.01890969 | 1 | 106182184;101747454 |
| BP | GO:0045087 | innate immune response | 2 | 15 | 0.0410827 | 1 | 101747386;426356 |
| BP | GO:0050778 | positive regulation of immune response | 1 | 3 | 0.06381246 | 1 | 101751261 |

Table S4. Immune-related GO enrichment statistics of IBV-vs-IBV/H9N2 DEGs

| Class | GO_ID | Term | DEGs in Term | All Gene in Term | P-value | Correct P-Value | DEGs list |
| --- | --- | --- | --- | --- | --- | --- | --- |
| BP | GO:0006955 | immune response | 32 | 103 | 2.79E-10 | 4.54E-07 | 420193;769069;395337;396495;100858035;419293;425935;395196;415652;395872;396216;769278;395451;420695;417534;427448;422654;100857191;424106;101752166;427187;426928;395485;428067;417464;427718;420696;417465;419276;428264;395551;417535 |
| BP | GO:0006954 | inflammatory response | 12 | 27 | 1.48E-06 | 0.00080514 | 100859040;772372;395196;420695;771173;101752245;420696;396370;417465;396451;428665;421219 |
| MF | GO:0008009 | chemokine activity | 11 | 24 | 2.82E-06 | 0.00133934 | 396495;415652;395872;395451;417534;422654;100857191;417464;417465;395551;417535 |
| MF | GO:0005125 | cytokine activity | 8 | 18 | 8.83E-05 | 0.02104611 | 100859040;395337;395196;396216;101752166;427718;428264;428665 |
| MF | GO:0004950 | chemokine receptor activity | 5 | 15 | 0.00857848 | 0.40747802 | 769278;420718;420695;395324;420696 |
| BP | GO:0002682 | regulation of immune system process | 2 | 2 | 0.00828074 | 0.51850185 | 395220;107052201 |
| BP | GO:0050727 | regulation of inflammatory response | 3 | 5 | 0.00654118 | 0.51850185 | 101747386;426356;420104 |
| BP | GO:0070098 | chemokine-mediated signaling pathway | 2 | 2 | 0.00828074 | 0.51850185 | 101752245;417465 |
| MF | GO:0016493 | C-C chemokine receptor activity | 3 | 6 | 0.01219912 | 0.52678026 | 769278;420695;420696 |
| BP | GO:0009607 | response to biotic stimulus | 1 | 10 | 0.61498625 | 1 | 422993 |
| MF | GO:0016494 | C-X-C chemokine receptor activity | 1 | 2 | 0.17375371 | 1 | 430652 |
| BP | GO:0042088 | T-helper 1 type immune response | 1 | 1 | 0.09101723 | 1 | 771173 |
| BP | GO:0045087 | innate immune response | 3 | 15 | 0.15049352 | 1 | 101747386;426356;421219 |

# Supplementary Figures


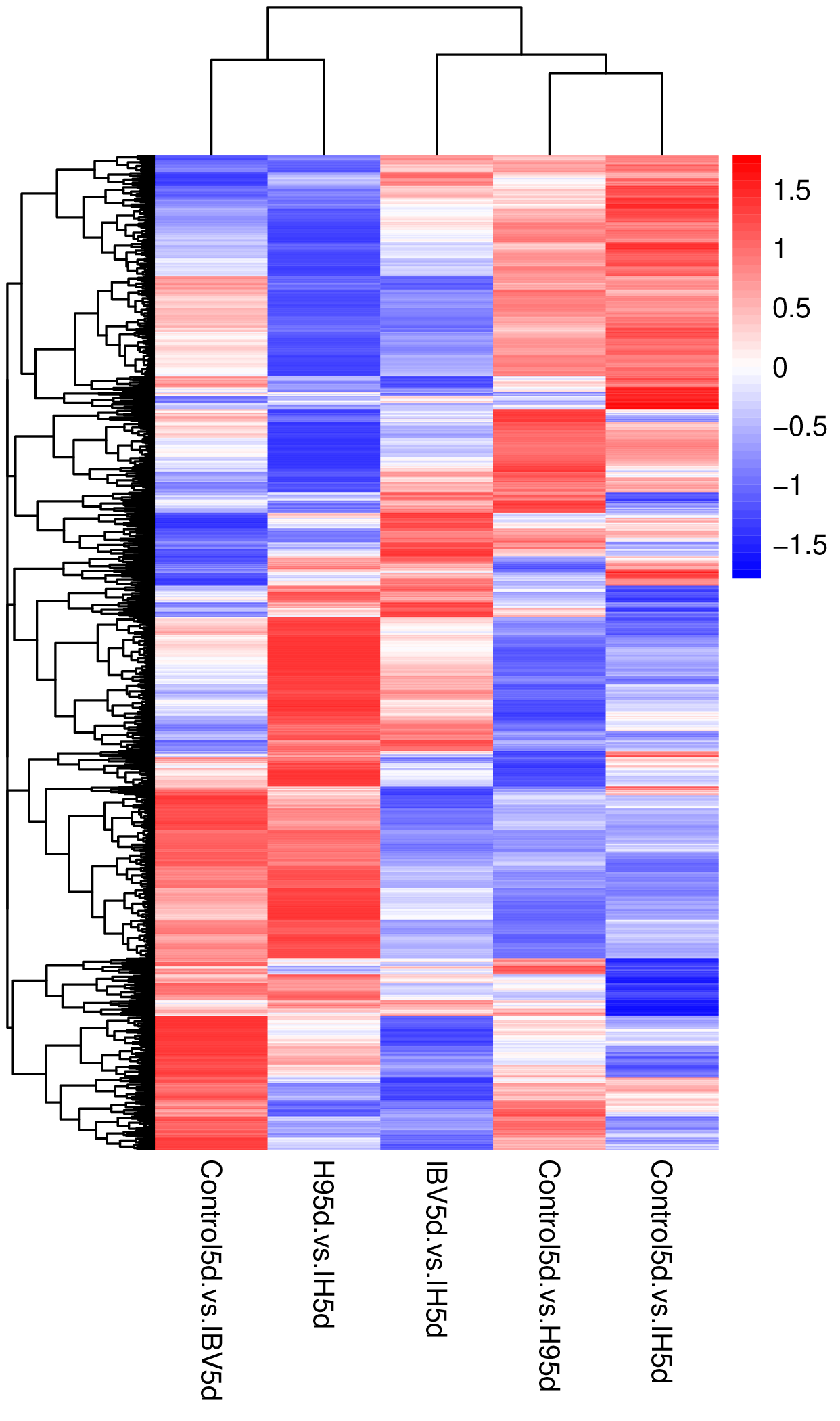


**Supplementary Figure 1.** Cluster diagram of deferentially expressed genes
